# Effect of drama training on self-esteem and personality strengths: A feasibility case control study of nidotherapy

**DOI:** 10.1177/00207640241239540

**Published:** 2024-03-20

**Authors:** Chelsea Mansell, Min Yang, Peter Tyrer

**Affiliations:** 1 Lincoln Medical School, University of Lincoln and Nottingham, UK; 2 Swinburne University of Technology, Melbourne, VIC, Australia; 3 West China School of Public Health, Sichuan University, Chengdu, China; 4 Division of Psychiatry, Imperial College, London, UK

**Keywords:** Drama therapy, self-esteem, personality strengths, case-control

## Abstract

**Background:**

Although there are many case reports and qualitative studies on the likely positive effects of drama on mental health there have been few quantitative studies with mentally ill patients.

**Aims:**

To assess the effect of drama training in patients receiving nidotherapy with a range of mental and personality disorders on changes in self-esteem and personality strengths over a 1-month period compared with two control groups, one with similar mental disorders and another without, who had similar assessments but no acting involvement.

**Method:**

A total of 19 patients were recruited from a mental health charity with current significant mental illness (active group: *n* = 6) (b) a control group of patients with current mental illness who were not involved in acting (*n* = 5), and an additional control group with no current mental illness (*n* = 8) The patients involved in drama were taking part in nidotherapy, an environmental intervention. Two self-rating scales, the Rosenberg Self-Esteem Scale (RSES) and Abbreviated Personality Strengths Scale (APSS) recorded changes in self-esteem and personality strengths at base-line and after 1 month in the participants. Random effects modelling was used to analyse the data.

**Results:**

The intervention group showed positive improvement in personality strengths (*p* = .009) compared to the control group that had no mental illness, and also improved more than the control group with mental illness but not to a significant degree (*p* = .16). Self-esteem recorded with the Rosenberg scale was lower in those in the acting group at baseline compared with the other two groups (*p* = .088) but after acting training improved by 29% to be equivalent to the control groups.

**Conclusions:**

Despite the limited numbers in this study, and the consequent inability to make firm conclusions about the efficacy of drama therapy as part of nidotherapy, the findings suggest that larger trials of this approach are feasible and worth exploring. .

## Introduction

Drama therapy and its related discipline, psychodrama, has long been used as both a method of understanding the complexities of mental illness and as therapeutic agents (Moreno, 1946; Ruddy & Dent-Brown, 2007). Despite this, much of the evidence of its efficacy has been impressionistic or qualitative in nature and there have been very few formal trials. One of the recent applications of drama therapy is its selection as a treatment in nidotherapy. Nidotherapy is the systematic collaborative manipulation of the environment to improve mental health in those with chronic mental illness (Tyrer et al., 2003). It is a person-centred approach whereby patients choose feasible environmental changes and follow a set of principles that increases autonomy, promotes agency and gives more power to change situations, often with the help of a nidotherapist as an environmental advocate (Tyrer & Tyrer, 2018). It is transdiagnostic and has been shown to be of value in two randomised trials (Ranger et al, 2009; Tyrer et al., 2017).

Drama therapy is linked to nidotherapy when it is felt in the initial environmental analysis that acting may have a role in effecting desired changes. It was employed as an aid to treatment when auditioning for performances of a new musical play, *The Battle of Stoke Field* (Tyrer, 2022) and in auditions for a radio play based on the journey of King James from Edinburgh to London in 1603 to take up the throne of England (Tyrer, 2018). The opportunity was taken to evaluate the impact of drama therapy in those receiving nidotherapy by comparing its outcomes with two control groups, one with a similar history of mental illness and another without such history.

## Method

### Study design

#### Active drama group

One of the environmental options for patients who have mental illness and ask for nidotherapy is to take part in drama therapy, but only if it is desired by the individuals concerned. All the patients recruited were from the registered charity, NIDUS-UK, a charity set up in 2013. Those who agreed to take part in the play were asked to read information about the project and sign a consent form that asked them to complete assessments at baseline and after 4 weeks. The six patients recruited to the study had the following long-term mental illnesses:

(1) (M) Chronic social anxiety disorder, leading to failure to engage in any occupational activity,
(2) (M) Persistent depression with suicidal thinking with only partial response to antidepressants,
(3) (M) Severe personality disorder following childhood trauma followed by self-imposed isolation and repeated suicidal behaviour,
(4) (M) Schizophrenia and comorbid drug misuse over 15 years with no settled occupation,
(5) (M) Severe depression following diagnosis of chronic heart disease that had major impact on ability to work
(6) (F) Drug and alcohol dependence associated with severe personality disorder

#### Control group 1

Five patients with chronic mental illness, three with personality disorder, one with alcohol dependence and one with a depressive disorder, who were not involved in the drama training were also included in the study.

#### Control group 2

Eight subjects who had no history of mental illness and who were not involved in drama training were also assessed in the study.

## Assessments

All participants taking part in the study completed a participation information sheet and consent form. They were asked to complete two scales at baseline that were felt to be most sensitive to change, the Rosenberg Self Esteem Scale (RSES) and the Abbreviated Personality Strengths Scale (APSS). The two rating scales were repeated after 4 weeks. The study tool place between October and December, 2022.

The Rosenberg Self Esteem Scale (Rosenberg, 1965) is the most widely used instrument to measure self-esteem (García et al., 2019). As higher self-esteem is also thought to be directly associated with a higher quality of life (Checa-Domene et al., 2022) positive changes have a major influence on well-being. Its 10 items have good reliability and consistency with a score ranging from 0 to 30 (Schmitt & Allik, 2005).

The Abbreviated Personality Strengths Scale developed from the Comprehensive Personality Assessment Schedule (CPAS), an extension of the Personality Assessment Schedule (Tyrer & Alexander, 1979), in which positive aspects of each personality trait were recorded. In a 30-year follow up study five personality strengths were identified from factor analysis – forceful consideration, emotional toughness, cautiousness, independence and discernment – and when these were present in sufficient strength they appeared to buttress patients against adversity and reduce suicidal behaviour (Yang, Tyrer, & Tyrer, 2022). The Abbreviated Scale addresses each of these strengths in an interview format.

## Statistical analysis

A descriptive analysis was performed of the two outcomes, presenting the means and standard deviation for each of the three comparison groups at the baseline and at the completion of training. We then tested difference among the three groups at the baseline and at the completion respectively using one-way ANOVA (two-sided), with the homogeneity of variance among the groups tested and confirmed before the ANOVA.

To examine the intervention effect, that is, the change in the outcome scores among the three groups, we used the random effects modelling approach. This approach takes account the repeated measures of each individual and handles missing data (two individuals missed data at completion in this case). This model includes three terms: group, time and interaction between group and time. The control group without mental illness was set as the reference group. The term *group* estimates the mean difference between the exposure / control without mental illness and the control with mental illness. The term *time* estimates mean difference between the two time points. The interaction term estimates mean difference in the outcome changes among groups, which tests for intervention effects. The generalise Ward test was used to test significance (two-sided) of the model estimates.

The packages IBM SPSS v26 and MLwiN v3.02 were used for the analysis.

## Results

A total of 19 subjects were assessed at baseline but two failed to complete data at 4 weeks; one of these developed a COVID-19 infection and could not continue in drama therapy. At baseline those with mental illness had lower self-esteem and fewer personality strengths than those in the second control group, with the lowest scores in the active drama group, an expected finding in view of the selection for nidotherapy (Table 1).

**Table 1.** Descriptive statistics of outcomes by comparison group by time.

	Active drama group	Control with CMI	Control without CMI	*p* value (ANOVA)
	*M* ± *SD*	*M* ± *SD*	*M* ± *SD*	
RSES				
Baseline	*n* = 6	*n* = 5	*n* = 8	.088
	14.33 ± 6.95	19.40 ± 5.55	21.63 ± 4.75	
After training	*n* = 5	*n* = 4	*n* = 8	.279
	18.40 ± 6.23	18.75 ± 6.50	23.00 ± 4.44	
Change	*n* = 5	*n* = 4	*n* = 8	
	3.00 ± 2.24	0.75 ± 2.63	1.37 ± 2.33	
APSS				
Baseline	*n* = 6	*n* = 5	*n* = 8	.323
	18.17 ± 5.71	23.00 ± 10.17	23.63 ± 4.90	
After training	*n* = 5	*n* = 4	*n* = 8	.959
	23.40 ± 6.11	22.75 ± 10.56	22.25 ± 5.39	
Change	*n* = 5	*n* = 4	*n* = 8	
	3.80 ± 4.60	0.75 ± 1.50	−1.38 ± 3.62	

After 4 weeks personality strengths and self-esteem had improved greatly in the active drama group and were roughly similar to the two control groups.

Although the means in the two outcomes did not show significant difference beyond chance both at the baseline and at the training completion, the larger change from the baseline to completion was more in the drama group than in the other two, and this justified testing the random effect models. This showed considerable variance across individuals overall but a significant degree of improvement in personality strengths with drama therapy (Table 2).

**Table 2.** Comparison in mean change of outcomes from the baseline to the completion of training among groups using random effects models.

	Estimate (*SE*)	P1	P2^a^
RSES			
Control without CMI (Ref.)	–		
Change in Intervention group	1.720 (1.35)	0.202	0.119
Change in control with CMI	−0.749 (1.45)	0.605	
Variance among individuals	28.896 (0.93)	**0.003**	
APSS			
Control without CMI (Ref.)	–		
Change in Intervention group	5.373 (2.05)	**0.009**	0.160
Change in control with CMI	1.987 (2.20)	0.367	
Variance among individuals	40.844 (14.42)	**0.005**	

Bold text indicates significant differences

*Note*. Interpretation: Both outcome measures had significantly large variation (*p* = .003 and .005) among individuals which was taken into account for the comparison of intervention effect among groups. The intervention group showed positive improvement in APSS (*p* = .009) compared to the control without CMI. The improvement seemed also higher than that of the control with CMI without reaching significance level (*p* = .160).

a Ward test *p* value in comparing mean change between the intervention and the control CMI.

## Discussion

This study was a feasibility study and it would be wrong to claim special efficacy for the drama intervention in view of the small numbers involved. The major limitations in addition to the numbers were the potential bias involved in selecting the subjects, particularly in the control group and the relatively short period of the study. The active and control groups were also not balanced for age and sex, but it is worthy of note that other studies have found no sex differences bias between male and females with the Rosenberg Scale (Mckay et al., 2014) and similar findings were shown with the Personality Strengths Scale (Yang, Tyrer, & Tyrer, 2022).

The choice of the Rosenberg and Personality Strengths scales as outcome measures seemed to have been justified by the results and these could be used again as they are relatively straightforward to complete and are closely linked to personality (Henriksen et al., 2017). It would be premature to recommend this approach generally as there are considerable difficulties in evaluating the benefits of psychodrama, almost the most complex of complex interventions (Campbell et al., 2000), and this is one of the reasons there are so few studies with control groups. Nonetheless, this small study offers an opportunity for similar evaluation including the collection of quantitative data and could be expanded into larger trials.

## Implications of the findings

This is the first study to evaluate the effects of drama as an element of nidotherapy, even though the option of dramatic performance has been part of the intervention for many years and has been used with patients taking part in public performances (Tyrer, 2017). Nidotherapy and other environmental therapies such as social prescribing (Hassan et al., 2020) are now involving group settings, including whole communities in which personality strengths are promoted (Jha et al., 2022), and as the performance of plays is essentially a group process this could be fostered as an important environmental option.

The results of the study also help to remove one of the canards attached to personality disorder; that it is untreatable and permanent. We do not pay enough attention to personality strengths. They are present in all, irrespective of any accompanying features that could be labelled as disorder, and explain why apparent personality disorder changes so often over the course of time (Yang, Tyrer, Johnson, & Tyrer, 2022).

## Conclusions

Although the small numbers in this study make firm conclusions speculative the marked gains in the acting group suggest this activity is highly efficacious in promoting self-esteem and boosting personality status. It suggests nidotherapy could be developed further in a group format.

## References

[bibr1-00207640241239540] Campbell M. Fitzpatrick R. Haines A. Sandercock P. Spiegelhalter D. Tyrer P. (2000). A framework for the design and evaluation of complex interventions to improve health. British Medical Journal, 321, 694–696. 10987780 10.1136/bmj.321.7262.694 PMC1118564

[bibr2-00207640241239540] Checa-Domene L. Luque De La Rosa A. Gavin-Chocano O. Torrado J. J. (2022). Students at risk: Self-esteem, optimism and emotional intelligence in post-pandemic times? International Journal of Environmental Research & Public Health, 19, 12499. 36231796 10.3390/ijerph191912499 PMC9566516

[bibr3-00207640241239540] García J. A. Y Olmos F. C. Matheu M. L. Carreño T. P. (2019). Self esteem levels vs global scores on the Rosenberg self-esteem scale. Heliyon, 5(3), e01378. 10.1016/j.heliyon.2019.e01378 PMC6434180 30963120

[bibr4-00207640241239540] Hassan S. M. Giebel C. Morasae E. K. Rotheram C. Mathieson V. Ward D. Reynolds V. Price A. Bristow K. Kullu C. (2020). Social prescribing for people with mental health needs living in disadvantaged communities: the Life Rooms model. BMC Health Services Research, 20, 19. 31906933 10.1186/s12913-019-4882-7 PMC6945402

[bibr5-00207640241239540] Henriksen I. O. Ranøyen I. Indredavik M. S. Stenseng F. (2017) The role of self-esteem in the development of psychiatric problems: A three-year prospective study in a clinical sample of adolescents. Child and Adolescent Psychiatry and Mental Health, 11, 68. 29299058 10.1186/s13034-017-0207-y PMC5747942

[bibr6-00207640241239540] Jha M. Barrett B. Brewin C. Bowker G. Harwood N. Jalil I. Crawford M. Phull J. Allen K. Duggan C. Yang M. (2022). Matching ICD-11 personality status to clinical management in a community team—The Boston (UK) Personality Project: Study protocol. Personality and Mental Health, 16, 130–137. 35474611 10.1002/pmh.1544

[bibr7-00207640241239540] Mckay M. T. Boduszek D. Harvey S. A. (2014). The Rosenberg Self-Esteem Scale: A bifactor answer to a two-factor question? Journal of Personality Assessment, 96, 654–60. 10.1080/00223891.2014.923436 24940657

[bibr8-00207640241239540] Moreno J. L. (1946). Psychodrama. Beacon House.

[bibr9-00207640241239540] Ranger M. Tyrer P. Miloseska K. Fourie H. Khaleel I. North B. Barrett B. (2009). Cost-effectiveness of nidotherapy for comorbid personality disorder and severe mental illness: Randomized controlled trial. Epidemiologia Psichiatrica Sociale, 18, 128–136. 19526744

[bibr10-00207640241239540] Rosenberg M. (1965). Society and the adolescent self-image. Princeton University Press.

[bibr11-00207640241239540] Ruddy R. A. Dent-Brown K. (2007). Drama therapy for schizophrenia or schizophrenia-like illnesses. Cochrane Database of Systematic Reviews, 2007, CD005378. 10.1002/14651858.CD005378.pub2 PMC12728680 17253555

[bibr12-00207640241239540] Schmitt D. P. Allik J. (2005). Simultaneous administration of the Rosenberg Self-Esteem Scale in 53 nations: exploring the universal and culture-specific features of global self-esteem. Journal of Personal and Social Psychology, 89, 623–642. 10.1037/0022-3514.89.4.623 16287423

[bibr13-00207640241239540] Tyrer P. (2017). The Death of King John. Blue Frog Publications.

[bibr14-00207640241239540] Tyrer P. (2018). The Watermeadow Mystery: The secret treasure at Hawton. Austin Macauley.

[bibr15-00207640241239540] Tyrer P. (2022). The Battle of Stoke Field. Lincoln, Impspired.

[bibr16-00207640241239540] Tyrer P. Alexander J. (1979). Classification of personality disorder. British Journal of Psychiatry, 135, 163–167. 10.1192/bjp.135.2.163 497619

[bibr17-00207640241239540] Tyrer P. Sensky T. Mitchard S. (2003). Principles of nidotherapy in the treatment of persistent mental and personality disorders. Psychotherapy and Psychosomatics, 72, 350–356. 14526138 10.1159/000073032

[bibr18-00207640241239540] Tyrer P. Tarabi S. A. Bassett P. Liedtka N. Hall R. Nagar J. Imrie A. Tyrer H. (2017). Nidotherapy compared with enhanced care programme approach training for adults with aggressive challenging behaviour and intellectual disability (NIDABID): Cluster-randomised controlled trial. Journal of Intellectual Disability Research, 61, 521–531. 28124397 10.1111/jir.12360

[bibr19-00207640241239540] Tyrer P. Tyrer H. (2018). Nidotherapy: Harmonising the environment with the patient (2nd ed.). Cambridge University Press.

[bibr20-00207640241239540] Yang M. Tyrer P. Tyrer H. (2022). The recording of personality strengths: An analysis of the impact of positive personality features on the long-term outcome of common mental disorders. Personality and Mental Health, 16, 120–129. 35532104 10.1002/pmh.1548 PMC9287073

[bibr21-00207640241239540] Yang M. Tyrer H. Johnson T. Tyrer P. (2022). Personality change in the Nottingham Study of neurotic disorder: 30 year cohort study. Australian and New Zealand Journal of Psychiatry, 56, 260–269. 34250845 10.1177/00048674211025624 PMC8866742

